# Natural history of falls in an incident cohort of Parkinson’s disease: early evolution, risk and protective features

**DOI:** 10.1007/s00415-017-8620-y

**Published:** 2017-09-25

**Authors:** Sue Lord, Brook Galna, Alison J. Yarnall, Rosie Morris, Shirley Coleman, David Burn, Lynn Rochester

**Affiliations:** 1 Human Movement Science, Institute of Neuroscience, Newcastle University Institute for Aging, Newcastle University, Newcastle upon Tyne, NE4 5PL UK; 2 NIHR Newcastle Biomedical Research Centre, Newcastle upon Tyne Hospitals NHS Foundation Trust and Newcastle University, Newcastle upon Tyne, UK; 3 School of Clinical Sciences, Auckland University of Technology, Auckland, New Zealand; 4 Newcastle upon Tyne Hospitals NHS Foundation Trust, Newcastle upon Tyne, UK; 5 UK and Industrial Statistics Research Unit, Newcastle University, Newcastle upon Tyne, UK; 6 Faculty of Medical Sciences, Newcastle University, Newcastle upon Tyne, UK

**Keywords:** Parkinson’s disease, Falls, Prognosis, Characteristics

## Abstract

The natural history of falls in early Parkinson’s disease (PD) is poorly understood despite the profound effect of falls on outcome. The primary aim of this study was to describe the natural history of falls, and characterise fallers over 54 months in 99 newly diagnosed people with PD. Seventy-nine (79.7%) participants fell over 54 months and 20 (20.3%) remained falls-naïve. Twenty six (26.2%) reported retrospective falls at baseline. Gait outcomes, disease severity and self-efficacy significantly discriminated across groups. Subjective cognitive complaints emerged as the only significant cognitive predictor. Without exception, outcomes were better for non-fallers compared with fallers at any time point. Between group differences for 54 month fallers and non-fallers were influenced by the inclusion of retrospective fallers and showed a broader range of discriminant characteristics, notably stance time variability and balance self-efficacy. Single fallers (*n* = 7) were significantly younger than recurrent fallers (*n* = 58) by almost 15 years (*P* = 0.013). Baseline performance in early PD discriminates fallers over 54 months, thereby identifying those at risk of falls. Clinical profiles for established and emergent fallers are to some extent distinct. These results reiterate the need for timely interventions to improve postural control and gait.

## Introduction

Falls aetiology in people with Parkinson’s disease (PD) is complex and multidimensional. Falls are associated with primary features (age, disease severity, gait and balance deficit, cognitive impairment), and secondary features that occur in response to falling (anxiety, reduced self-efficacy, weakness and loss of mobility) [1]. To date, the strongest predictor of a future fall is a prior fall [2], and clinical fall assessment is typically triggered when falls are established and not prior to their occurrence. Although pragmatic, this approach is limited and important to recognise because effective falls management becomes more challenging when secondary features are established. Ideally, management of falls risk should begin early and aim to prevent or at least delay the onset of falls. The early natural history of falls and emerging risk factors is, however, poorly understood.

Responding to these concerns we reported on risk factors for falls in a cohort of falls-naïve, newly diagnosed people with PD [3]. We assessed falls over 36 months from diagnosis using monthly falls diaries alongside a comprehensive battery of motor, cognitive and clinical measures. Slow gait speed, decreased stance time and Hoehn and Yahr III emerged as significant baseline predictors with 92% sensitivity and 62% specificity. Risk of falling was almost eight times higher for people presenting with these characteristics. Cognition did not feature as a risk factor despite recent evidence supporting a clear role in falls prediction [4–6] and in promoting effective falls intervention programmes [7] possibly because the level of cognitive impairment did not reach a threshold of effect.

We now extend these findings by taking a more nuanced and detailed approach to the data. Previous research has identified the need for personalised fall prevention strategies [1], but until the drivers of falls (both primary and secondary features) are more fully understood this is difficult to achieve. Characteristics that identify falls risk may differ for established and new fallers, and also influence fall frequency and the circumstances in which falls occur [8, 9]. In this study, we document evolution of falls over 54 months from diagnosis in an incident cohort of PD. We examine baseline characteristics of emergent fallers and non-fallers over 54 months to identify features that precipitate and alternatively protect from falls. We identify baseline features that discriminate fallers from falls-naïve at 54 months from diagnosis; identify protective features in people who remain falls-naïve; explore baseline differences in falls risk in single versus recurrent falls; and explore the impact of a previous fall on fall risk characteristics.

## Methods

### Participants

The methodology has been described in full in the earlier publication and elsewhere [3, 10–12], and brief details only are given here. Participants were recruited into ICICLE-GAIT within 4 months of diagnosis. This is a collaborative study with ICICLE-PD, an incident cohort study (Incidence of Cognitive Impairment in Cohorts with Longitudinal Evaluation—Parkinson’s disease) conducted between June 2009 and December 2011. Participants were optimally medicated and tested ‘on’ medication for clinical, gait and cognitive measures, which were defined as 1 h after medication. All testing took place at the Clinical Ageing Research Unit, Newcastle University. The study was approved by the Newcastle and North Tyneside Research Ethics Committee and all participants gave informed consent.

#### Clinical assessment

Disease severity was measured using the movement disorder society (MDS)-revised unified Parkinson’s disease rating scale (UPDRS) and Hoehn and Yahr (H&Y) stage [13]. PIGD scores and motor phenotype were determined according to the MDS-UPDRS revision [14]. Balance self-efficacy was measured using the activities balance self confidence scale [15], fatigue with the multidimensional fatigue inventory [16] and depression with the geriatric depression scale (GDS) [17]. The presence or absence of freezing of gait (FoG) was calculated from the new FOG score [18] Levodopa equivalent daily dose (LEDD) scores were calculated for each patient [19]. Systolic and diastolic blood pressures were recorded in the supine position after 10 min rest and then 3 min after standing. Orthostatic hypotension was defined as a 20 mmHg fall in systolic BP and/or a 10 mmHg in diastolic BP [20].

#### Gait assessment and outcomes

Gait was assessed using a 7 m long × 0.6 m wide instrumented walkway (Platinum model Gaitrite, software version 4.5, CIR systems, United States of America). Participants were instructed to walk at their comfortable walking pace for 2 min around a 25 m oval circuit under single and dual task conditions. We measured 16 independent gait characteristics that conform to a model of gait that has been validated in PD and comprises five independent domains (pace, variability, rhythm, asymmetry and postural control) [21].

#### Cognitive assessment and outcomes

Cognitive tests have been described in full elsewhere, as noted above. Briefly, global cognition was measured with the Montreal Cognitive Assessment (MoCA) [22]. Attention was assessed with the cognitive drug research (CDR) battery [23] and tests from the CANTAB battery measured visual memory and executive function [24]. Visuospatial function was assessed with the first item of the MoCA which included clock drawing, cube copying and a short version of the Trail Making Test B. Two items from the Parkinson’s disease non-motor-symptoms questionnaire [25] were used to record subjective cognitive complaint: participants were asked whether in the last month they had experienced problems remembering things that have happened recently or forgetting to do things (item 12) and difficulty in concentrating or staying focused (item 15).

#### Falls diaries

A fall was defined as ‘unintentionally coming to rest on the ground or other lower surface without being exposed to overwhelming external force or a major internal event’ [26] and falls were recorded prospectively using PROFANE recommendations. Falls diaries were sent out on a monthly basis with a pre-paid return envelope in which participants were asked to record if they had fallen in the past month. If so, they were prompted to provide the date and time of each fall as well as location, preceding activity, perceived cause, position in which they landed on the floor and mode of recovery. All falls reported in the diaries were followed up with a telephone call from a senior research physiotherapist (DM, HH) to verify information and rectify any missing data. Reasons for non-returned diaries were ascertained by follow-up telephone calls, and participants were encouraged to continue if appropriate. If diaries for single fallers were incomplete, their data could not be used to establish frequency because their status was unclear. Those who identified as retrospective fallers were reported as a ‘single faller’ at baseline. We classified falls using the falls-related activities classification [8, 9] to discern the type of fall experienced by single fallers.

### Data analysis

Falls occur along a continuous timeline, but for the purposes of this study we annotated falls at discrete and evenly spaced intervals that corresponded with follow-up assessments. Data from monthly diaries were inspected and a five-point ordinal scale was used to reflect falls status: 0 = falls-naïve at 54 months; 1 = retrospective faller (self-reported faller at study entry); 2 = new faller between baseline and 18 months; 3 = new faller between 18 and 36 months; 4 = new faller between 36 and 54 months. We first described baseline characteristics for each group and examined between group differences. We then selected outcomes to represent the broad scope of characteristics described in Table 1 and conducted two analyses. The first compared fallers versus non-fallers at 54 months with inclusion and then exclusion of baseline fallers; and the second compared single versus recurrent fallers. For the latter analysis, fall frequency was determined by inspection of falls diaries and only participants with completed diaries over 54 months were included. Finally, we analysed proportion of fallers at discrete intervals over 54 months for four outcomes which were shown to be significantly different between groups: Hoehn and Yahr staging, subjective cognitive complaint, and gait speed and gait variability. These four outcomes were dichotomised around median scores for the total cohort (*n* = 99). Because of non-normal distribution of data and small sample size, we used non-parametric tests to describe and examine between group differences for all analyses. Given the exploratory nature of the analysis we accepted an alpha level of *P* < 0.05.

**Table 1:** Baseline demographic and clinical characteristics of retrospective fallers and new fallers at 18 month intervals over 54 months

	Retrospective (baseline) fallers	Prospective fallers			Non-fallers	*P*
	(*n* = 26)	18 m (*n* = 30)	36 m (*n* = 17)	54 m (*n* = 6)	(*n* = 20)	
Age (years)	66.9 (13.2)	71.9 (13.0)	70.2 (15.9)	70.9 (11.8)	67.6 (16.9)	0.863
BMI	25.5 (6.4)	26.3 (6.1)	25.5 (7.2)	26.1 (5.4)	26.8 (5.6)	0.935
Male, *n* (%)	13 (50)	23 (76.6)	9 (52.9)	5 (83.3)	16 (80)	0.078
Levodopa prescribed, *n* (%)	13 (50)	7 (23.3)	5 (29.4)	1 (16.6)	6 (30)	0.216
LEDD (mg day^−1^)	180.0 (285.0)	100.0 (57.5)	137.5 (200.0)	100.0 (255.5)	135.0 (165.0)	0.174
Dopamine agonist, *n* (%)	9 (34.6)	9 (30)	7 (41.1)	1 (16.6)	8 (40)	0.660
Disease severity						
H&Y score, *n* (%) I, II, III	2 (7.6), 18 (69.2), 6 (23.0)	6 (20), 12(60), 12 (60)	6 (35.2), 10(58.8), 1 (5.8)	0 (0), 6 (100), 0 (0)	8 (40), 11(55), 1(5)	**0.002**
Motor phenotype, *n* (%) (PIGD, ID, TD)	14 (53.8), 3 (11.5), 9 (34.6)	17 (56.6),4 (13.3), 9(30)	9 (52.9), 1 (5.8), 7 (41.1)	1(16.6), 1 (16.6), 4 (66.6)	6 (30), 1 (5), 13 (65)	**0.015**
PIGD score (0–4)	0.60 (0.8)	0.60 (0.4)	0.60 (0.5)	0.40 (0.4)	0.40 (0.4)	**0.022**
Tremor score (0–4)	0.75 (0.50)	0.55 (0.55)	0.64 (0.60)	0.77 (0.53)	0.72 (0.53)	0.233
UPDRS II (0–52)	12.0 (8)	11.0 (5)	11.0 (10)	9.0 (10)	7.0 (8)	0.144
UPDRS III (0–132)	29.0 (15.7)	27.0 (12.5)	26.0 (15.5)	23.0 (17.0)	19.5 (11.7)	0.058
FOG, *n* (%)	5 (19.2)	1 (3.3)	1 (5.8)	0 (0)	2 (10.0)	0.370
NFOG score (0–28; higher worse)	0 (0)	0 (0)	0 (0)	0 (0)	0 (0)	0.726
Orthostatic hypotension, *n* (%)	6 (23.0)	5 (16.6)	1 (5.8)	3 (50.0)	2 (10.0)	0.493
GDS (0–15; higher worse)	3.0 (3)	3.0 (3)	2.0 (2)	2.0 (3)	2.0 (1.0)	0.140
ABC score (0–100; higher better)	69.0 (44.6)	89.3 (26.3)	93.5 (20.9)	93.6 (17.1)	96.2 (18.9)	**<0.001**
Total fatigue (20–100; higher worse)	64.0 (17)	49.0 (19)	46.0 (21)	51.0 (19)	37.5 (34)	**0.000**
Cognitive outcomes						
Years of education	12.0 (6)	11.5 (4)	11.0 (8)	13.0 (4)	11.5 (6)	
MoCA (0–30)	24.5 (7.2)	25.0 (5.2)	26.0 (4.7)	27.5 (3.5)	27.0 (5.5)	0.232
MoCA visuospatial (Item 1) (0–5)	4.0 (2.0)	4.0 (2.0)	5.0 (1.7)	5.0 (0.25)	5.0 (1.0)	0.205
Subjective cognitive complaint, *n* (%)	24 (92.3)	19 (63.3)	10 (58.8)	3 (50.0)	8 (40)	**<0.001**
PoA (mean SRT, CRT, digit vigilance)	1362 (260)	1357 (156)	1246(336)	1360(300)	1287(154)	0.227
PRM (mean correct latency)	2165 (1510)	2072 (646)	2180 (902)	2023 (660)	1987 (753)	0.604
SRM (mean correct latency)	2024 (1434)	2128 (892)	2335 (525)	2089 (924)	1971 (758)	0.965
OTS (mean correct latency)	22,145 (18,658)	22,452 (15,615)	20,500 (11,122)	12,802 (6144)	19,039 (13,998)	0.338
OTS (mean choices correct)	1.2 (0.21)	1.2 (0.30)	1.3 (0.37)	1.5 (0.55)	1.4 (0.50)	0.328
Motor outcomes						
Single leg stance (s) (higher better)	6.7 (20.5)	9.9 (19.8)	5.3 (15.6)	18.5 (11.2)	13.2 (23.4)	0.092
Timed chair stand (s) (higher worse)	13.6 (4.9)	14.6 (5.9)	12.4 (4.7)	14.6 (4.5)	13.3 (5.4)	0.595
Gait outcomes						
Pace domain						
Step velocity (m/s)	1.05 (0.27)	1.03 (0.24)	1.11 (0.36)	1.12 (0.24)	1.21 (0.14)	**0.021**
Step length (m)	0.590 (0.11)	0.615 (0.14)	0.621 (0.12)	0.658 (0.14)	0.657 (0.10)	**0.044**
Swing time variability (ms)	20.0 (11.6)	16.9 (8.70	15.3 (6.3)	15.4 (2.6)	13.7 (7.2)	
Rhythm domain						
Mean step time (ms)	551.0 (63.8)	579.6 (82.1)	533.5 (59.0)	569.5 (91.3)	537.5 (42.4)	0.215
Mean swing time (ms)	381.7 (32.3)	391.4 (51.1)	399.6 (49.1)	395.1 (62.9)	383.0 (40.8)	0.421
Mean stance time (ms)	723.7 (98.5)	749.1 (123.4)	695.7 (100.1)	752.0 (104.5)	699.9 (50.0)	0.074
Variability domain						
Step velocity variability (m s^−1^)	0.057 (0.020	0.051 (0.01)	0.047 (0.01)	0.048 (0.01)	0.048 (0.01)	**0.039**
Step length variability (m)	0.024 (0.01)	0.022 (0.00)	0.018 (0.00)	0.016 (0.00)	0.020 (0.01)	**0.044**
Step time variability (ms)	22.2 (11.5)	19.0 (8.4)	14.5 (8.5)	15.8 (2.2)	15.3 (8.0)	**0.016**
Stance time variability (ms)	26.1 (16.9)	23.3 (8.7)	15.9 (10.3)	16.6 (4.2)	16.5 (7.3)	**0.002**
Asymmetry domain						
Swing time asymmetry (ms)	12.3 (8.3)	11.8 (21.8)	13.4 (26.0)	19.0 (12.0)	7.6 (10.1)	0.082
Step time asymmetry (ms)	14.7 (20.1)	13.3 (27.3)	19.8 (20.4)	20.0 (8.0)	10.4 (15.2)	0.460
Stance time asymmetry (ms)	12.6 (9.9)	11.1 (20.4)	11.1 (27.0)	16.7 (9.7)	10.1 (7.80	0.453
Postural control domain						
Step length asymmetry (m)	0.022 (0.03)	0.014 (0.02)	0.020 (0.04)	0.030 (0.02)	0.019 (0.02)	0.494
Mean step width (m)	0.105 (0.05)	0.086 (0.05)	0.086 (0.03)	0.085 (0.03)	0.080 (0.04)	0.806
Step width variability (m)	0.018 (0.00)	.0.018 (0.00)	0.019 (0.00)	.0.016 (0.00)	0.017 (0.00)	0.988

Significant values are indicated in bold

Data presented as the group median (IQR) unless otherwise stated

All comparisons using Kruskal–Wallis test apart from binary outcomes [Chi-square (*χ* ^2^) test]

*LEDD* levodopa equivalent daily dose, *PIGD* postural instability and gait disorder subscale UPDDRS, *UPDRS III* united Parkinson’s disease rating scale, *NFOG* new freezing of gait questionnaire, *ABCs* activities balance confidence-specific scale, *GDS* geriatric depression scale, *MoCA* Montreal cognitive assessment, *MCR* motor cognitive risk, *PoA* power of attention, *PRM* pattern recognition memory, *SRM* spatial recognition memory, *OTS* one touch stocking of Cambridge

## Results

One hundred and twenty-one people with incident PD were recruited into ICICLE-GAIT. Two participants were re-diagnosed and two did not participate in the falls study, leaving a total of 117 participants. Eighteen participants whose falls status was unknown withdrew from the falls study over the 54 months, with data available for 99 participants. Of these, 26 (26.2%) reported at least one fall in the past year (retrospective faller). From baseline to 18 months 30 (30.3%) prospective fallers were recorded, from 18 to 36 months 17 new fallers (17.1%), and from 36 to 54 months 6 (6.0%) new fallers. By 54 months 79 (79.7%) of the total cohort had fallen and 20 (20.3%) were still falls-naive. Prospective data for fall frequency were available for 65 participants with complete diaries to 54 months. Seven participants (10.7%) were single fallers, and 58 (89.3%) recurrent. Participants who fell did not revert to non-fallers.

Table 1 describes characteristics of fallers and non-fallers and between group differences. Descriptive data shows that outcomes were almost universally better for the 20 non-fallers compared with fallers at any time point. Over 54 months, disease severity, gait, and outcomes that broadly represent self-efficacy (including subjective cognitive complaint) were the strongest discriminative features for new fallers at each time point. However, the spread of H&Y scores suggests some inconsistencies. For example, fallers at 36 months who presented as H&Y III comprised only 1 of a total of 17 (5.6%). A similar discrepancy occurred in retrospective fallers, with only 6 of the 26 (23%) classified as H&Y II. Although the proportion of new fallers increased rapidly from baseline to 18 months especially for those with H&YIII, around a third of H&Y I had also fallen by this stage. Median PIGD score was 0.60 for fallers at baseline through to 36 months, lowering to 0.40 for fallers at 54 months and for non-fallers. Features of gait from pace and variability domains discriminated across groups, with non-fallers presenting with the fastest gait. Freezing of gait was most evident in retrospective fallers. Subjective cognitive complaint was the only cognitive variable to significantly discriminate across groups with 92% of baseline fallers reporting subjective complaint, gradually lowering to 40% for 54 month fallers. The influence of retrospective fallers on falls status at 54 months is illustrated in Table 2, which shows stronger between group (fallers versus non-fallers) discrimination for a broader range of outcomes when retrospective fallers are included in analysis. Differences in single leg time, step velocity and PIGD are the only variables retained as significant when retrospective fallers are excluded from analysis. Table 3 shows selected baseline characteristics for seven participants who comprised the single faller cohort, who were compared with recurrent fallers (*n* = 58) at 54 months. Single fallers were significantly younger by 15 years, and presented with higher MoCA visuospatial scores which trended towards significance (*P* = 0.05). When single falls were classified according to pre-fall event [9] three falls occurred during advanced activity (one whilst hiking and two whilst walking outdoors); three during combined movement (stair descent); and one during postural transition (rising from sitting to stand).

**Table 2:** Comparison of baseline characteristics of all fallers and non-fallers at 54 months, including and excluding retrospective (baseline) fallers

	54-month falls cohort including retrospective fallers		*P*	54-month falls cohort excluding retrospective fallers		*P*
	Fallers (*n* = 72)	Non-fallers (*n* = 23)^a^		Fallers (*n* = 53)	Non-fallers (*n* = 20)	
Age	70.0 (12.8)	66.2 (17.2)	0.380	71.6 (12.0)	67.6 (16.9)	0.451
BMI	26.2 (6.7)	26.2 (5.0)	0.555	26.1 (6.7)	26.8 (5.6)	0.951
UPDRS III	26.5 (12.5)	21.9 (12.0)	**0.043**	26.0 (13.5)	19.5 (11.7)	0.083
PIGD	0.60 (0.6)	0.40 (0.4)	**0.005**	0.60 (0.5)	0.40 (0.4)	**0.021**
H&Y stage I, II, III, *n* (%)	13 (18.0) 42 (58.4), 17 (23.6)	9 (39.1) 13 (56.5), 1 (4.4)	**0.034**	12 (22.6), 28 (52.8), 13 (24.6)	8(40.0) 11(55.0), 1(5.0)	0.106
Single leg time	7.8 (18.9)	15.3 (23.5)	**0.020**	8.0 (19.3)	13.2 (23.4)	**0.036**
ABCs	87.9 (27.3)	95.6 (11.2)	**0.029**	92.0 (23.9)	96.2 (18.9)	0.202
MoCA total	26.0 (4.0)	27.0 (6.0)	0.140	26.0 (4.7)	27.0 (5.5)	0.379
MoCA visuospatial	5 (2.0)	5 (1.0)	0.388	5.0 (2.0)	5 (1.0)	0.877
PoA	1346 (204)	1282 (187)	**0.075**	1348 (189)	1287 (154)	0.212
Step velocity	1.08 (0.26)	1.21 (0.15)	**0.001**	1.09 (0.25)	1.21 (0.14)	**0.006**
Stance time SD	21.9 (11)	16.4 (5.4)	**0.012**	21.3(11.0)	16.5 (7.3)	0.071

Significant values are indicated in bold

Data presented as the group median (IQR) unless otherwise stated

Total sample size for 54 m falls cohort (*n* = 95) is smaller than the total cohort described in Table 1 (*n* = 99) because four participants (three retrospective non-fallers and one retrospective faller) were included in baseline data but did not participate in the prospective study

All Mann–Whitney other than H&Y (*χ* ^2^ test)

^a^ Non-fallers at 54 months include three retrospective fallers

**Table 3:** Comparison of baseline characteristics of single versus recurrent fallers over 54 months

	Single fallers (*n* = 7)	Recurrent fallers (*n* = 58)	P*
Age	56.9 (15.7)	71.7 (11.9)	**0.013**
BMI	25.2 (10.6)	26.3 (5.9)	0.719
UPDRS III	26.0 (8.0)	26.5 (11.7)	0.857
PIGD	0.60 (0.4)	0.60 (0.6)	0.528
H&Y stage I, II, III, *n* (%)	2 (28.5), 4 (57.2), 1 (14.3)	10 (17.2), 32 (55.2), 16 (27.6)	0.588
Single leg time	22.2 (22.3)	6.9 (18.5)	0.423
ABCs	90.8 (28.1)	85.4 (30.0)	0.315
MoCA total	27.0 (2.5)	25.0 (5.0)	0.082
MoCA visuospatial	5 (5.0)	4.0 (2.0)	0.052
PoA	1244 (302)	1349 (187)	0.186
Step velocity	0.97 (0.5)	1.06 (0.21)	0.642
Stance time SD	18.7 (22.4)	22.2(10.8)	0.612

Significant value is indicated in bold

Data presented as the group median (IQR) unless otherwise stated

* All Mann–Whitney other than H&Y (Chi-square test)

**Fig. 1:**
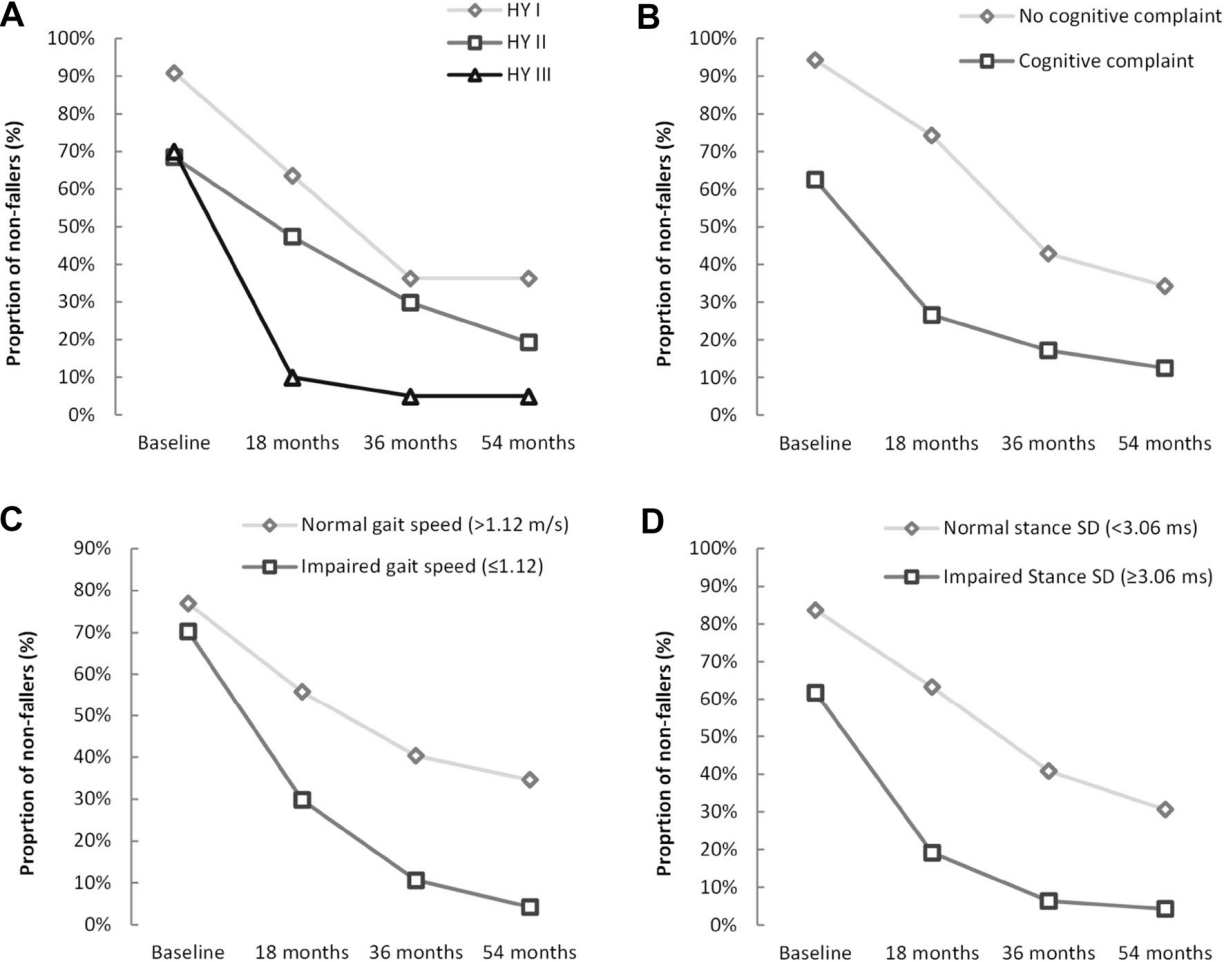
Proportion of non-fallers over 54 months excluding retrospective fallers: **a** H&Y groups, **b** subjective cognitive complaint, **c** gait speed, and **d** stance time variability

Figure 1 shows proportion of non-fallers at each time point for four variables. Figure 1a shows an early, steep decline in proportion of non-fallers for H&Y III followed by a plateau (*P* = 0.004; Chi-square test); Fig. 1b shows a similar line for participants with and without subjective cognitive complaint but a greater proportion of fallers across all time points for participants who reported a complaint at baseline (*P* = 0.017; Chi-square test); Fig. 1c, d show comparable trajectories for gait speed and stance time variability, with faster speed and lower variability protective of falls, both of which significantly discriminated across groups (*P* = 0.019 and *P* = 0.013, respectively, Kruskal–Wallis test).

## Discussion

This study is, to our knowledge, the first to describe the natural history of falls in people with PD over an extended period of time and relate this to prognostic falls risk. Participants experienced their first fall soon after diagnosis, with most new fallers appearing before 36 months. Over the 54 months only seven participants retained single faller status, suggesting that sporadic falls are uncommon.

The detailed characterisation of participants who fell and those who did not inform early management of falls risk. Overall our results show that disease severity and gait discriminate new fallers, along with aspects of self-efficacy which are more evident when retrospective fallers are included in analysis. Deficits in postural control (the hallmark feature of H&Y III) are evident even in very early PD [27] and these results along with differences in single leg stance confirm the critical role of postural control to falls. Whilst these results appear obvious, a more nuanced consideration shows the distribution of H&Y scores varies across the 54 months, suggesting that characteristics other than disease severity contribute to falls. This point to the complexity of falls risk, which is the interaction of multiple factors. Our findings are broadly consistent with a large body of prognostic falls research, some of which has also been carried out in falls-naïve cohorts using robust methodology to ascertain falls status [28, 29]. An important distinction is that participants from these earlier studies had moderately advanced disease (average duration for both studies 6 years), by which time secondary features such as deconditioning are likely to influence results. For example, Almeida [28] identified self-reported disability status as the strongest significant predictor of all falls, suggesting a role for self-efficacy which may not appear earlier.

We expected cognition (global cognition, attention and executive dysfunction) to emerge as discriminative measures given their powerful role in modifying falls and the presence of early cognitive impairment in PD. Attention is a powerful modifier of gait [12] and we anticipated a stronger link in this cohort. Cognitive impairment is a significant risk factor for falls in older adults and in PD with recent evidence suggesting its effect may be underestimated [30]. A recent study exploited the role of cognition to falls in PD using a dual task paradigm delivered via a virtual reality platform which yielded superior results compared with treadmill training alone [7]. The results may partly reflect the advanced status of the cohort where the association between cognition and falls is likely to be stronger. Our results did not confirm a significant role for cognition, although we may have been underpowered to detect differences. Descriptively, scores for global cognition (MoCA) and power of attention were worse for early fallers, as was visuospatial function. Subjective cognitive complaint discriminated across groups, however, the stability of the measure has to our knowledge not been verified, and cross-sectional data from the larger ICICLE-PD study indicates that follow-up scores for subjective cognitive complaint show an inconsistent pattern with many participants reverting from a positive score at baseline to a negative score at 18 months [31]. A more discreet understanding of cognition in falls trajectory and its contribution relative to motor dysfunction is required help inform strategies for early intervention. Of interest is a recent study reporting comparable fall frequency for people with PD of 6 years average duration with and without mild cognitive impairment [30], suggesting that cognition may emerge as an important feature later than this. We anticipated that cognitive reserve (resilience against age-induced cognitive change), measured by years of education, would exert a protective effect but this was not the case with highly comparable outcomes across the falls spectrum.

More broadly, we confirmed that retrospective fallers were more globally impaired at baseline than falls-naïve participants. Inclusion of retrospective fallers biases findings towards more generic features, some of which may reflect secondary change occurring in response to falls or age-related features. For example, scores for balance self-efficacy (ABCs) and fatigue were worse for retrospective fallers compared with fallers at all time points. Between group differences were overall not as strong when retrospective fallers were excluded. These findings have important implications for understanding falls risk and prioritising treatment. Predictors of falls will be different when established fallers are included in analysis, which is the most common methodology. Their inclusion potentially mask important findings.

Single fallers were significantly younger and presented with better visuospatial function when compared with recurrent fallers. Classification of each single fall event [9] further informed about the nature of fall risk for this group and the likelihood of recurrence. We published 12-month falls classification data for this cohort [9] and argue for the need to document falls in this broader context so that more useful inferences about prognosis can be made.

Limitations to this study include the sample size. There were only six new fallers at 54 months, which compromised variance in scores. We were underpowered to detect all discriminatory features, indicated by some *P* values which denote a trend towards significance. Counter to this argument is that the study design involved a comprehensive test battery which in turn provides in-depth descriptive data. Future analysis will review falls trajectory over a longer time period and identify prognostic features.

In conclusion, new fallers emerge consistently over 54 months in newly diagnosed PD, although most occur early after diagnosis and become recurrent. New fallers can be categorized by baseline performance, especially for measures of disease severity and motor function. The contribution of cognition to falls status is less emphatic in this early group. Retrospective fallers present with more global features of decline, some of which may be age-related. These findings help guide clinical decision-making and emphasize the need for early interventions that target in particular gait and postural control deficit.
